# The Histone Acetyltransferase p300 Regulates the Expression of Pluripotency Factors and Odontogenic Differentiation of Human Dental Pulp Cells

**DOI:** 10.1371/journal.pone.0102117

**Published:** 2014-07-09

**Authors:** Tong Wang, Huijuan Liu, Yanyang Ning, Qiong Xu

**Affiliations:** 1 Guanghua School of Stomatology & Guangdong Provincial Key Laboratory of Stomatology, Sun Yat-sen University, Guangzhou, China; 2 Hefei Stomatological Hospital, Hefei, China; The University of Adelaide, Australia

## Abstract

p300 is a well-known histone acetyltransferase (HAT) and coactivator that plays vital roles in many physiological processes. Despite extensive research on the involvement of p300 in the regulation of transcription in numerous cell lines, the roles of this protein in regulating pluripotency genes and odontogenic differentiation in human dental pulp cells (HDPCs) are poorly understood. To address this issue, we investigated the expression of OCT4, NANOG and SOX2 and the proliferation and odontogenic differentiation capacity of HDPCs following p300 overexpression. We found that p300 overexpression did not overtly affect the ability of HDPCs to proliferate. The overexpression of p300 upregulated the promoter activity and the mRNA and protein expression of NANOG and SOX2. The HAT activity of p300 appeared to partially mediate the regulation of these factors; indeed, when a mutant form of p300 lacking the HAT domain was overexpressed, the promoter activity and expression of NANOG and SOX2 decreased relative to p300 overexpression but was greater than in the control. Furthermore, we demonstrated that the mRNA levels of the odontogenic marker genes dentine matrix protein-1 (*DMP-1*), dentin sialophosphoprotein (*DSPP*), dentin sialoprotein (*DSP*), osteopontin (*OPN*) and osteocalcin (*OCN*) were significantly decreased in HDPCs overexpressing p300 cultured under normal culture conditions and increased in HDPCs inducted to undergo odontogenic differentiation. This finding was further confirmed by measuring levels of alkaline phosphatase (ALP) activity and assessing the formation of mineralized nodules. The HAT activity of p300 had no significant effect on odontogenic differentiation. p300 was recruited to the promoter regions of *OCN* and *DSPP* and might be acting as a coactivator to increase the acetylation of lysine 9 of histone H3 of *OCN* and *DSPP*. Collectively, our results show that p300 plays an important role in regulating the expression of key pluripotency genes in HDPCs and modifying odontogenic differentiation.

## Introduction

The transcription coactivator p300, which encodes a 300 kDa protein, is a member of the lysine acetyltransferase 3 (KAT3) family of histone acetyltransferase (HAT) transcriptional coactivators and was originally identified as a protein that was bound to the adenoviral E1A [1]. p300 is a large nuclear protein found in most mammalian cells, and it plays an important role in the regulation of genes controlling cell proliferation, apoptosis, differentiation, the cell cycle and DNA repair [2]. Numerous transcription factors can interact with p300 either directly or indirectly through coactivators to stimulate the transcription of specific genes [3]. p300 has been shown to possess acetyltransferase activity [4], which enables it to influence chromatin structure by modifying nucleosomal histones. The acetylation of amino-terminal lysine residues on histone tails loosens chromatin, thereby increasing the accessibility of the DNA to transcriptional factors [5]. This modification of histones opens the chromatin and facilitates the direct recruitment of specific transcriptional regulators that regulate gene expression [6].

OCT4, NANOG and SOX2 have been identified as core factors that cooperatively form a positive regulatory circuitry for regulating and maintaining the self-renewal and pluripotency of embryonic stem (ES) cells [7], [8]. OCT4, NANOG and SOX2 are also expressed in somatic stem cells, and the overexpression of these pluripotent factors in somatic stem cells results in increased proliferation and differentiation potential in these cells [9], [10]. Several reports have provided strong evidence for the critical role of histone acetyltransferases such as p300, Tip60, Mof and Gcn5 in regulating the expression of genes of the core transcriptional network in mouse ES cells [11]–[14]. p300 is involved in regulating the transcription of *OCT4, NANOG* and *SOX2* target genes and thus regulates the transcriptional network in ES cells [15]. p300 can be recruited to genomic sequences bound by OCT4, NANOG or SOX2, and the depletion of any of these factors reduces the binding of p300 at such sites [14]. p300 acetylates histones at the distal regulatory region of NANOG and is therefore directly involved in modulating NANOG expression in differentiating mouse ES cells [11]. p300 may also interact with the transactivation domain of SOX2 and thereby synergistically coactivate SOX2 and OCT3/4. p300 can acetylate the DNA-binding domain of SOX2 and thus enhance global acetylation in mouse ES cells [16].

Dental pulp cells (DPCs) are composed of ectodermic and mesenchymal components containing neural crest-derived mesenchymal progenitors endowed with plasticity and multipotency. The ability of these cells to form colonies is greater than that of MSCs from the bone marrow [17]. DPCs are easily obtained from extracted teeth, possess high proliferative ability, and can be reprogrammed into induced pluripotent stem (iPS) cells at relatively high rates [18], [19]. OCT4, NANOG and SOX2 have been detected in the early passages of cells derived from the dental pulp and might be markers of differentiation of DPCs [20], [21]. p300 can be detected during murine tooth development [22]. However, the expression profile of p300 in HDPCs has not been described, and it is not known whether p300 is involved in regulating the expression of these pluripotency factors or promoting odontogenic differentiation in human dental pulp cells.

In this study, we explored the expression pattern of p300 in HDPCs. We then developed HDPC/p300 and HDPC/p300-ΔHAT cell lines. We show that p300 upregulates the expression of NANOG and SOX2 but not OCT4; we also show that the inherent HAT activity of p300 is required for this regulation. The overexpression of p300 can improve the odontogenic differentiation potentiality of HDPCs that have been induced to undergo odontogenic differentiation; however, it has no impact on the proliferation of HDPCs.

## Results

### p300 expression levels in wild-type HDPCs

**Figure 1:**
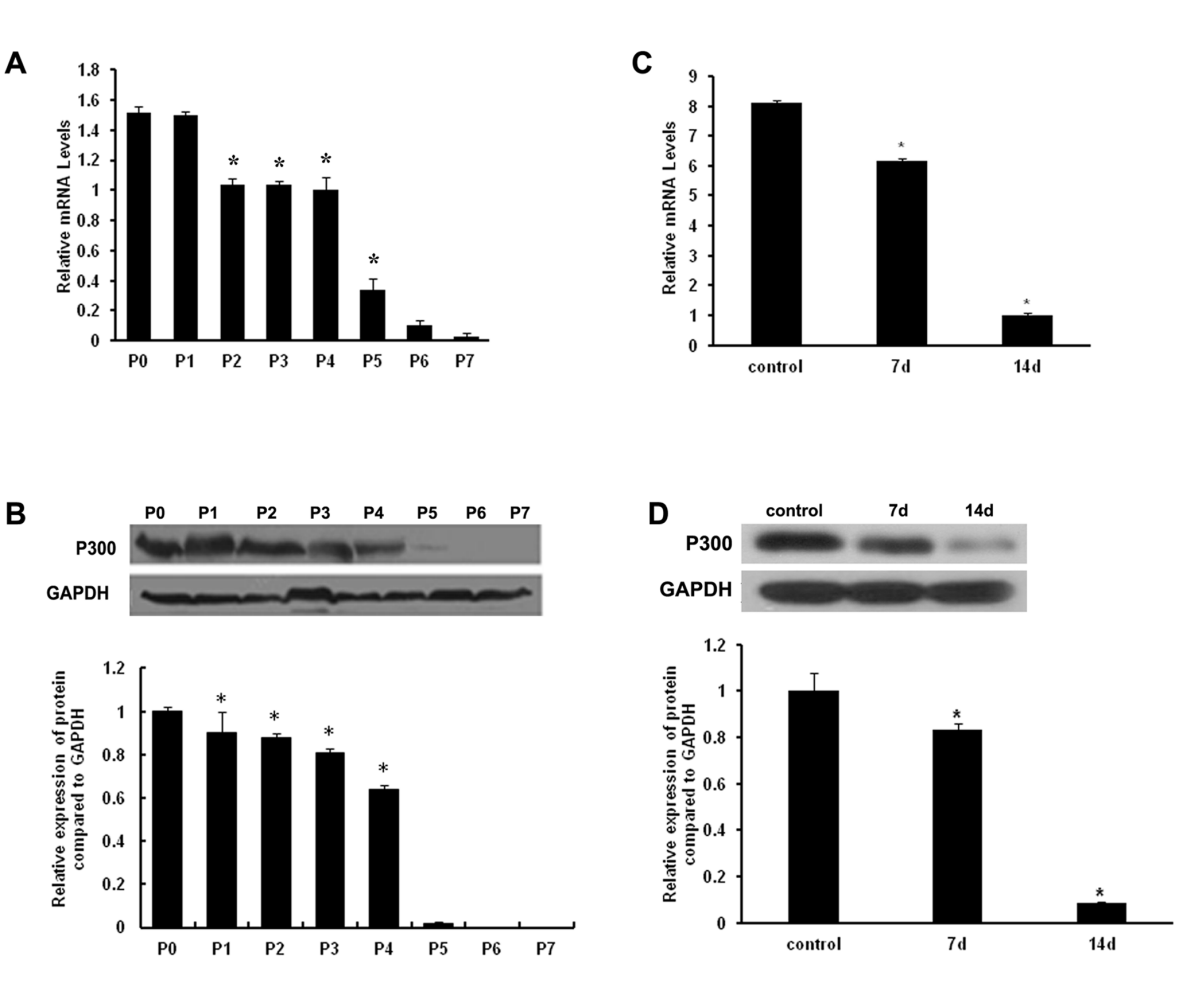
The expression pattern of p300 in wild-type HDPCs. (A) Real-time qPCR was performed to measure p300 mRNA levels in wild-type primary HDPCs and in HDPCs serially passaged one to seven times. The mRNA level of each product was normalized to GAPDH mRNA levels. (B) The protein expression level of p300 was assessed by western blotting analysis (right panel) and densitometric evaluation (left panel; expressed as the ratio of p300 to GAPDH). (C) p300 mRNA levels were measured in HDPCs undergoing odontoblastic differentiation. (D) p300 protein levels were assessed in HDPCs undergoing odontoblastic differentiation by western blotting analysis (right panel) and densitometric evaluation (left panel; expressed as the ratio of p300 to GAPDH). All results are presented as the means ± SD of three independent experiments. Procedures were performed as described in the text (n = 3). * Statistically significant difference relative to the control, *P*<0.05.

First, we examined the levels of p300 mRNA and protein in wild-type primary HDPCs and in HDPCs serially passaged one to seven times. As shown in Figures 1A and 1B, p300 mRNA and protein could be detected in the HDPCs. p300 levels were highest in the primary HDPCs and decreased in subsequent passages. p300 levels were almost undetectable in HDPCs passaged seven times. Thus, our data show that p300 mRNA and proteins are present in wild-type HDPCs.

We next examined the expression of p300 in HDPCS undergoing odontogenic differentiation (Figure 1C, D). Odontogenic differentiation was induced in HDPCs for 7 and 14 days; real-time qPCR and western blotting showed that p300 mRNA and protein levels decreased during this period and that p300 levels were lower after 14 days than after 7 days. These findings suggest that the expression of p300 is down-regulated during the induction of odontogenic differentiation.

### Stable overexpression of p300 and p300-ΔHAT in HDPCs

**Figure 2:**
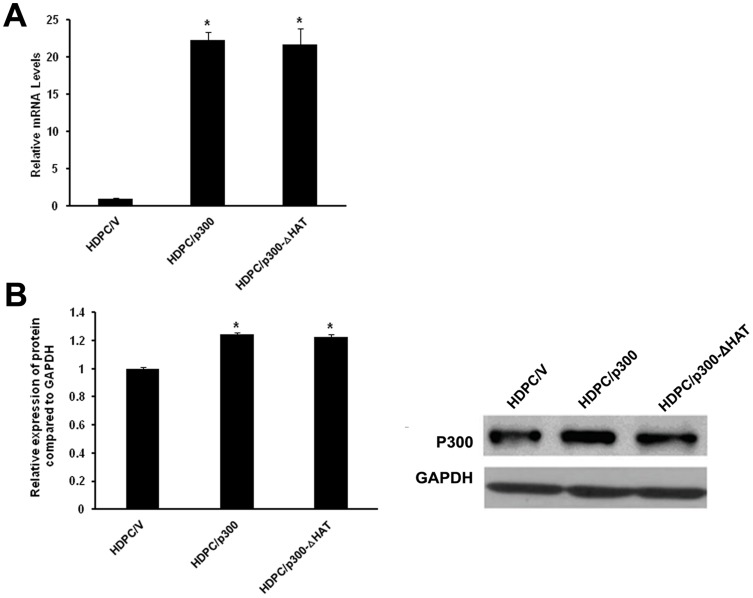
The stable overexpression of p300 and p300-ΔHAT in HDPCs. (A) real-time qPCR was performed to measure the relative levels of p300 and p300-ΔHAT mRNA after the transduction with lentiviral vectors. The level of each product was normalized to GAPDH mRNA levels. (B) Protein expression levels of p300 and p300-ΔHAT were assessed by western blotting analysis (right panel) and densitometric evaluation (left panel; expressed as ratio to GAPDH). The expression of GAPDH served as a control. All results are presented as the means ± SD of three independent experiments. Procedures were performed as described in the text (n = 3). * Statistically significant differences relative to the control, *P*<0.05.

To investigate whether HDPCs respond to stimulation with p300 or p300-ΔHAT, we stably transduced HDPCs with lentiviral vectors overexpressing p300 or p300-ΔHAT or with a control vector. GFP expression was measured 3 days after the transduction using fluorescence microscopy. We then examined the expression levels of p300 or p300-ΔHAT with real-time qPCR analysis and western blotting. We found that the levels of p300 and p300-ΔHAT mRNA and protein were higher in p300-overexpressing and p300-ΔHAT-overexpressing HDPCs (HDPC/p300, HDPC/p300-ΔHAT) than in the negative control cells (HDPC/V). The expression of GAPDH did not vary significantly among the groups, suggesting that p300 and p300-ΔHAT were stably expressed in the HDPCs (Figure 2A, 2B).

### Effects of p300 on the proliferation of HDPCs

**Figure 3:**
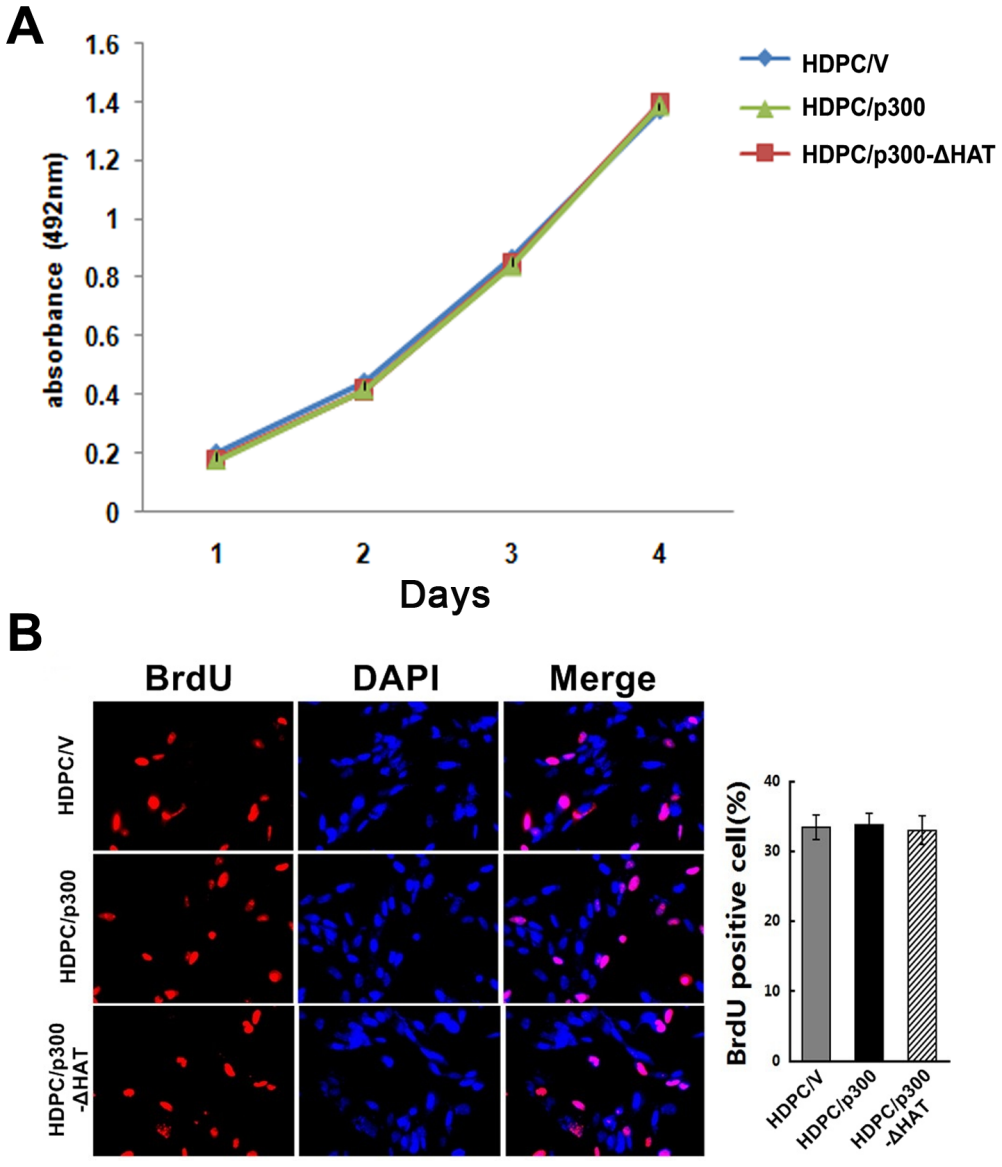
The effect of p300 on the proliferation of HDPCs. (A) Cell growth curves of HDPC/p300, HDPC/p300-ΔHAT and HDPC/V cells were constructed with the results of the CCK8 assay. The growth curves showed that p300 does not have a significant effect on the proliferation of HDPCs. (B) The BrdU assay revealed no significant differences in the amount of DNA synthesized by HDPC/p300, HDPC/p300-ΔHAT and HDPC/V cells. Newly synthesized DNA is stained red by BrdU and nuclei are stained blue by DAPI. Procedures were performed as described in the text (n = 3). All results are presented as the means ± SD of three independent experiments.

The growth rates of HDPC/p300, HDPC/p300-ΔHAT and HDPC/V cells cultured in normal culture media were compared. Cell proliferation was measured at 1, 2, 3 and 4 days with the CCK8 assay. As shown in Figure 3A, the three types of HDPCs grew at similar rates over the 4 days of observation, and no significant differences in growth rates were observed among the three groups. To confirm these results, we tested cell proliferation with a BrdU assay. As shown in Figure 3B, no significant differences in DNA synthesis were observed among the three groups. These results suggest that p300 may not play an important role in enabling the proliferation of HDPCs.

### p300 regulates the expression of key pluripotency factors in HDPCs

**Figure 4:**
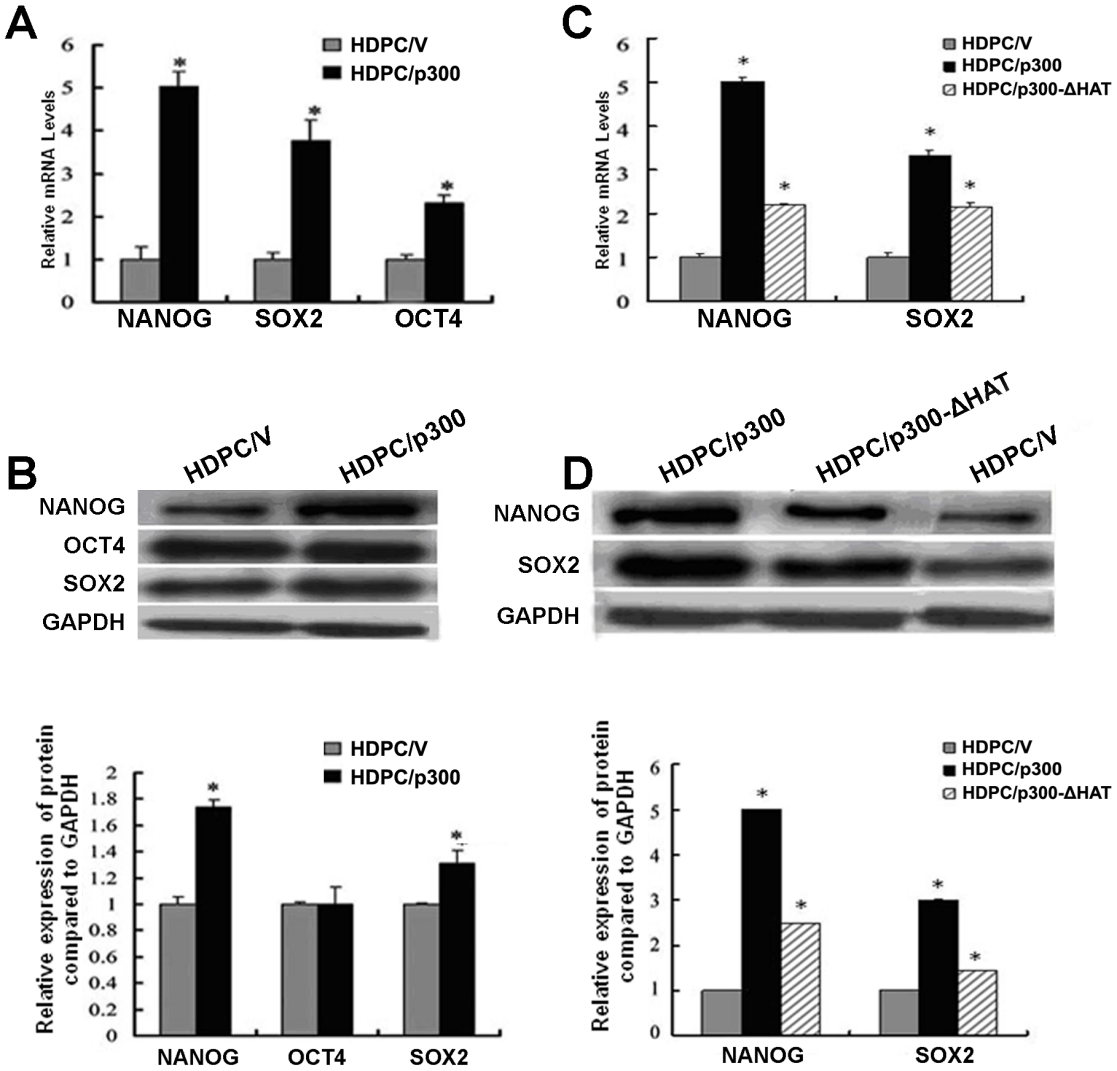
p300 regulates the expression of OCT4, NANOG and SOX2, whereas HAT mutant p300 suppresses the expression of NANOG and SOX2. (A) Real-time qPCR estimation of the relative endogenous mRNA levels of *OCT4, NANOG* and *SOX2* in HDPC/p300 and HDPC/V cells. The mRNA levels of each product were normalized to the mRNA levels of GAPDH. (B) Western blotting analysis (right panel) and densitometric evaluation (left panel; expressed as the ratio of protein levels to GAPDH levels) measuring the levels of OCT4, NANOG and SOX2 proteins in HDPC/p300 and HDPC/V cells. The expression of GAPDH was used as an internal control. (C) Measurement of the relative endogenous mRNA levels of *NANOG* and *SOX2* in HDPC/V, HDPC/p300 and HDPC/p300-ΔHAT cells using real-time qPCR. The mRNA levels of each product were normalized to GAPDH mRNA levels. (D) The results were further confirmed by western blotting (right panel) and densitometric evaluation (left panel; expressed as ratio to GAPDH). GAPDH was used as an internal control. All results are expressed as the means ± SD of three independent experiments. Procedures were performed as described in the text (n = 3). * Statistically significant difference relative to the control, *P*<0.05.

To evaluate the effects of the overexpression of p300 on the endogenous expression of the key pluripotency genes *OCT4, NANOG* and *SOX2* in HDPCs, we performed real-time qPCR and western blotting analyses to determine whether mRNA and protein levels of these genes differed in the HDPC/p300 and HDPC/V groups. Real-time qPCR demonstrated that *OCT4, NANOG* and *SOX2* mRNA levels were increased in the p300-transfected HDPCs (Figure 4A). As shown in Figure 4B, the patterns of change in the protein and mRNA levels of these pluripotency factors differed. Whereas the protein levels of NANOG and SOX2 were upregulated in cells with p300 overexpression, the protein level of OCT4 was not significantly altered.

We then evaluated whether the intrinsic HAT activity of p300 was necessary for the activation of NANOG and SOX2 expression in HDPCs. As shown in Figure 4C, real-time qPCR revealed that the deletion of the p300 HAT domain, which abrogated the HAT activity of p300, partially suppressed its ability to promote the transcription of the *NANOG* and *SOX2* genes. However, significantly higher levels of protein and gene expression were observed in the p300-ΔHAT mutant than in the control. We next confirmed the effects of the wild-type p300 and p300-HAT mutants by western blotting analysis (Figure 4D); as was the case with mRNA levels, we found that the levels of NANOG and SOX2 proteins were decreased in cells lacking the HAT domain. These results suggest that functional HAT activity is partially required for the modulation of NANOG and SOX2 mRNA and protein expression by p300.

### p300 enhances the activities of the NANOG and SOX2 promoters

**Figure 5:**
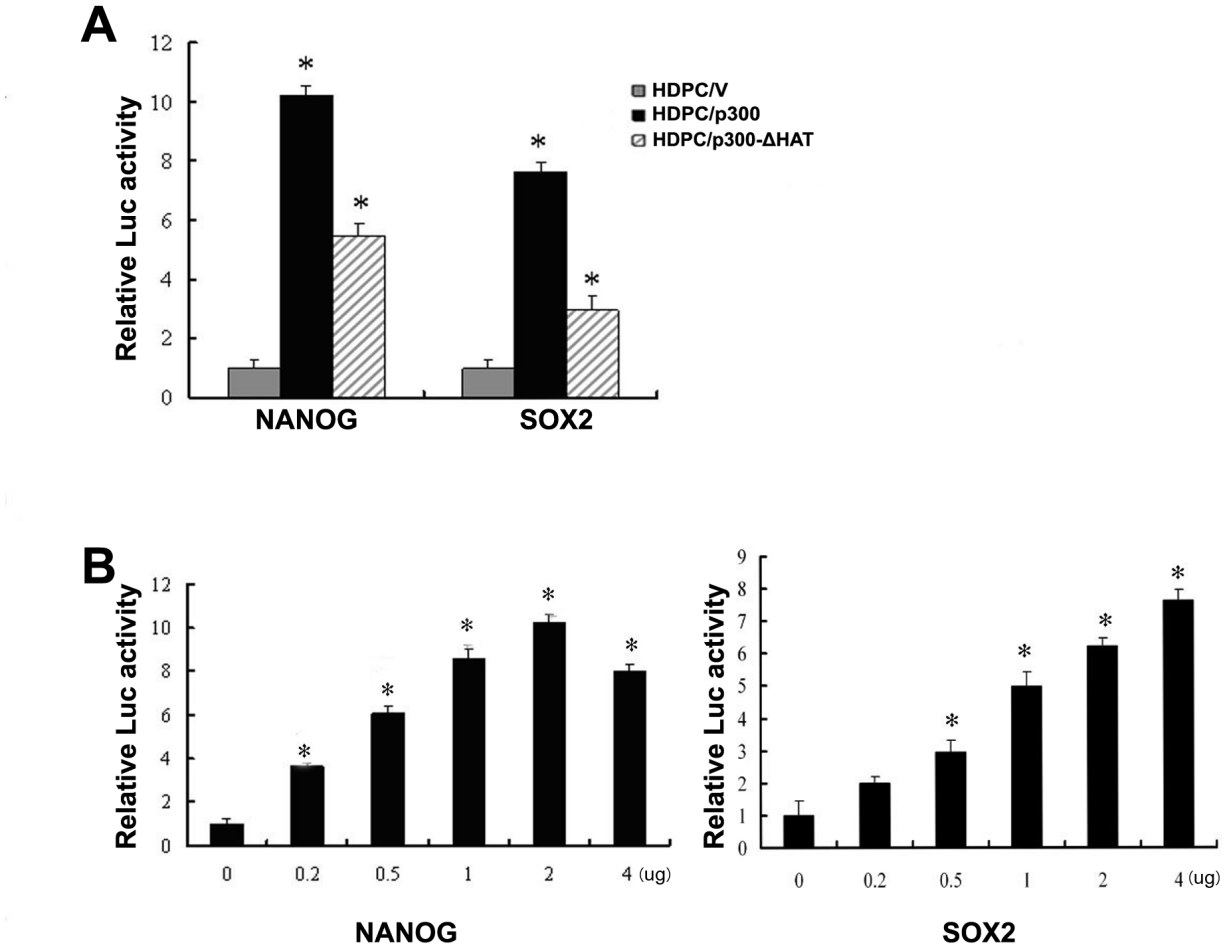
p300 increases the activities of the *NANOG* and *SOX2* promoters. (A) HDPCs were transiently transfected with 500 ng of the *NANOG* or *SOX2* promoter construct and 5 µg of the p300 or p300-ΔHAT expressing vector. Cells were harvested and the activities of the promoter were measured after 24 hours of transfection. (B) HDPCs were transiently transfected with 500 ng of the *NANOG* or *SOX2* promoter construct and increasing amounts (0–5 µg) of the p300-expressing vector. After 24 hours of transfection, the cells were harvested and the activities of the promoters were measured. Procedures were performed as described in the text (n = 3). All results are presented as the means ± SD of three independent experiments. * Statistically significant difference relative to the control, *P*<0.05.

Co-transfection studies were performed to determine whether p300 is involved in regulating the activities of the *NANOG* and *SOX2* promoters. First, the constructed promoter reporter plasmid and the exogenous p300 or p300-ΔHAT expression plasmid were transiently co-transfected into HDPCs. The cells were harvested and the relative luciferase activity was measured. As shown in Figure 5A, *NANOG* and *SOX2* promoter activities were increased by p300 overexpression; in contrast, stimulation with the p300-ΔHAT plasmid resulted in much lower levels of *NANOG* and *SOX2* promoter activities. However, significantly higher levels of *NANOG* and *SOX2* promoter activities were observed in the p300-ΔHAT mutant than in the control. The HDPCs were next transiently transfected with varying amounts of the p300 expression plasmid and the reporter plasmid. Figure 5B shows that p300 was able to promote the transcriptional activities of the *NANOG* and *SOX2* promoters in a dose-dependent manner.

### Effects of p300 on odontoblastic differentiation and the mineralization potential of HDPCs following the induction of odontoblastic differentiation

Our results showed that the overexpression of p300 in HDPCs upregulated the expression of NANOG and SOX2, the markers of cell “stemness”. We next determined whether p300 induced the HDPCs to be more stem-cell-like (and therefore less differentiated) and whether p300 played a role in regulating the odontoblastic differentiation potential of HDPCs.

**Figure 6:**
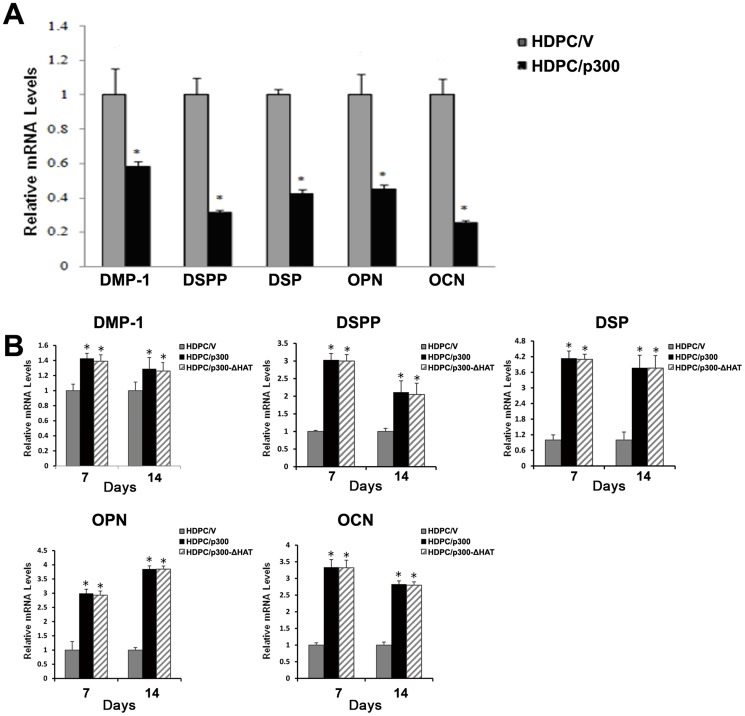
The overexpression of p300 regulates the expression of odontoblastic marker genes in HDPCs in normal growth medium or odontoblastic induction medium. (A) Real-time qPCR examination of the relative endogenous mRNA levels of *DMP-1, DSPP, DSP, OPN* and *OCN* in cells with overexpressed p300 and in negative control cells cultured under normal culture conditions. The mRNA levels of each product were normalized to GAPDH mRNA levels. (B) The overexpression of p300 increases the expression of odontoblastic marker genes in HDPCs induced to differentiate. Total RNA was extracted from the induced cells. The endogenous mRNA expression of *DMP-1, DSPP, DSP, OPN* and *OCN* was measured by real-time qPCR on days 7 and 14. GAPDH was used as an internal control. All results are presented as the means ± SD of three independent experiments. Procedures were performed as described in the text (n = 3). * Statistically significant difference relative to the control, *P*<0.05.

To test whether p300 can induce the HDPCs to become less differentiated, we measured the mRNA levels of critical marker genes of odontoblastic differentiation (*DMP-1, DSPP, DSP, OPN* and *OCN*) in p300-transfected HDPCs cultured under normal culture conditions using an empty vector as a control. The results indicated that mRNA levels of *DMP-1, DSPP, DSP, OPN* and *OCN* were significantly reduced (Figure 6A) and demonstrate that the overexpression of p300 can suppress the expression of odontoblastic marker genes and induce HDPCs to be less differentiated.

It has been reported that the odontoblastic differentiation ability of HDPCs can be improved by culturing the HDPCs in odontoblastic induction medium for several days [24], [25]. To investigate whether p300 plays a role in regulating the odontoblastic differentiation potential of HDPCs, we examined the expression of odontoblastic differentiation markers, the activity of ALP, and the formation of mineralized nodules in HDPC/p300 and HDPC/p300-ΔHAT cells induced to undergo odontoblastic differentiation. The cells were cultured in a differentiation induction medium for 7 and 14 days. The mRNA levels of *DMP-1, DSPP, DSP, OPN* and *OCN* genes were measured by real-time qPCR (Figure 6B). The alterations in the expression levels of *DMP-1, DSPP, DSP, OPN* and *OCN* were similar at all time points in HDPC/p300 and HDPC/p300-ΔHAT cells. Significantly higher levels of mRNA of these genes were observed in HDPC/p300 and HDPC/p300-ΔHAT cells than in HDPC/V cells on days 7 and 14 of odontoblastic induction. These results indicate that p300 can upregulate the expression of odontoblastic marker genes when HDPCs are induced to differentiate, even in the absence of HAT activity.

**Figure 7:**
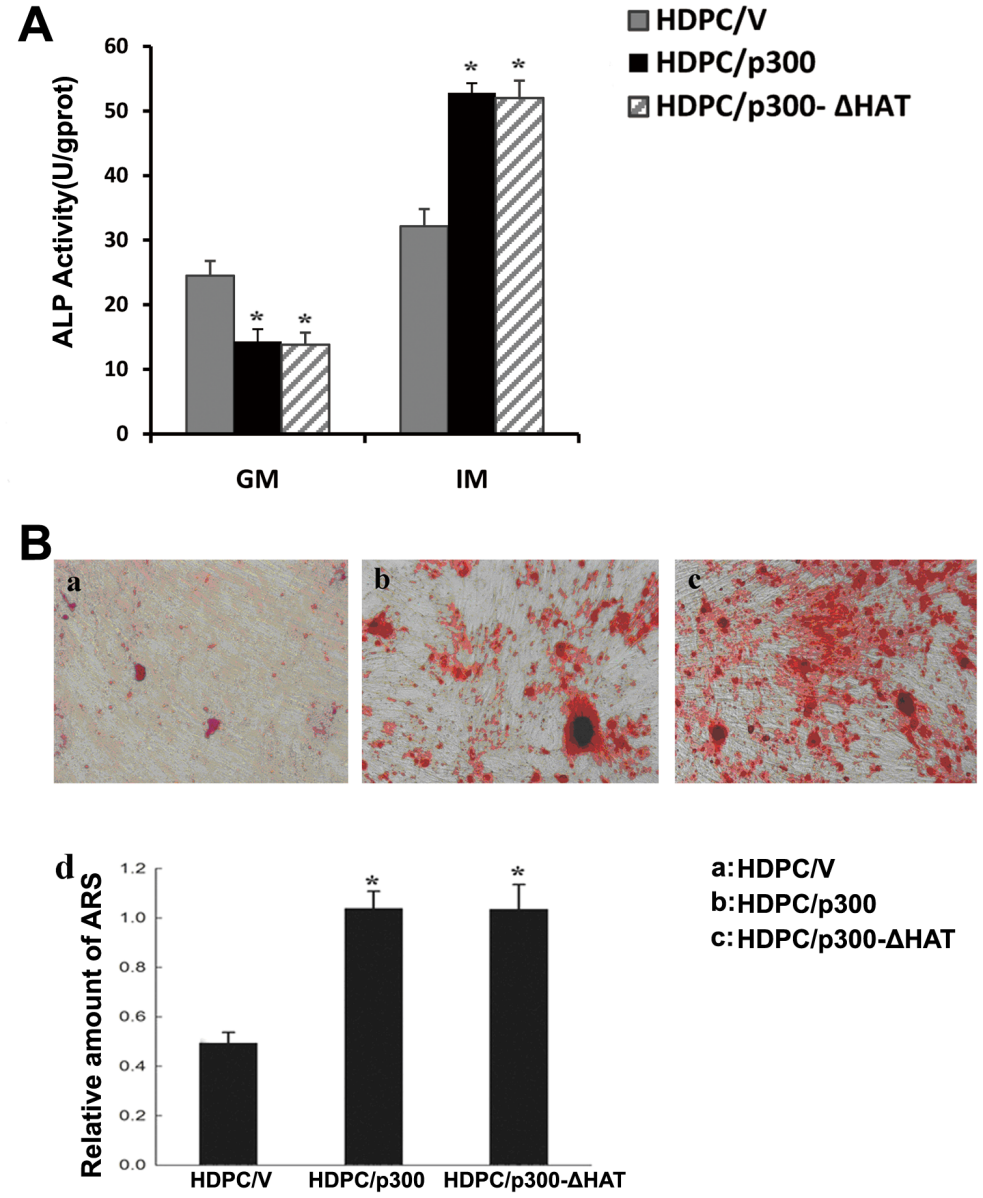
The overexpression of p300 increases ALP activity and mineral formation in HDPCs. (A) HDPC/V, HDPC/p300 and HDPC/p300-ΔHAT cells were cultured for 3 days in normal growth medium or odontoblastic induction medium, and the ALP activity in these cells was measured. GM, normal growth medium; IM, odontoblastic induction medium. (B) The effect of p300 on the formation of mineralized nodules in HDPCs cultured in odontoblastic induction medium, as analyzed by alizarin red S staining on day 21(×100). a: HDPC/V; b: HDPC/p300; c: HDPC/p300-ΔHAT. Scale bar, 100 µm. d: The histogram shows the quantification of mineralization by densitometry and reveals that remarkable decreases in mineralization occurred in HDPC/p300 and HDPC/p300-ΔHAT cells relative to control cells. All results are presented as the means ± SD of three independent experiments. Procedures were performed as described in the text (n = 3). * Statistically significant difference relative to the control, *P*<0.05.

We next measured ALP activity (an early marker of osteoblasts and odontoblasts) in cells not exposed to odontoblastic induction and in cells cultured in odontoblastic induction medium for 3 days. As shown in Figure 7A, HDPC/V cells had higher levels of ALP activity under normal culture conditions. However, when the cells were induced to differentiate for 3 days, HDPC/p300 and HDPCs/p300-ΔHAT cells had greater levels of ALP activity than the control cells. These results indicate that the overexpression of p300 results in increased ALP activity in HDPCs induced to differentiate.

The formation of nodules is considered to be an important feature of mineralization in HDPCs. To identify the effect of p300 on the formation of mineralized nodules, cells were cultured in differentiation medium for 21 days and alizarin red S staining was used to assess the formation of calcified nodules. On day 21, mineralized nodules were detected by microscopy; the overexpression of p300 and p300-ΔHAT accelerated the formation of nodules to a similar extent. The number of mineralized nodules was significantly higher in HDPC/p300 and HDPC/p300-ΔHAT cells than in HDPC/V cells (Figure 7B).

### p300 is recruited to the promoter of OCN and DSPP and increases the acetylation of lysine 9 of histone H3 of OCN and DSPP promoters

**Figure 8:**
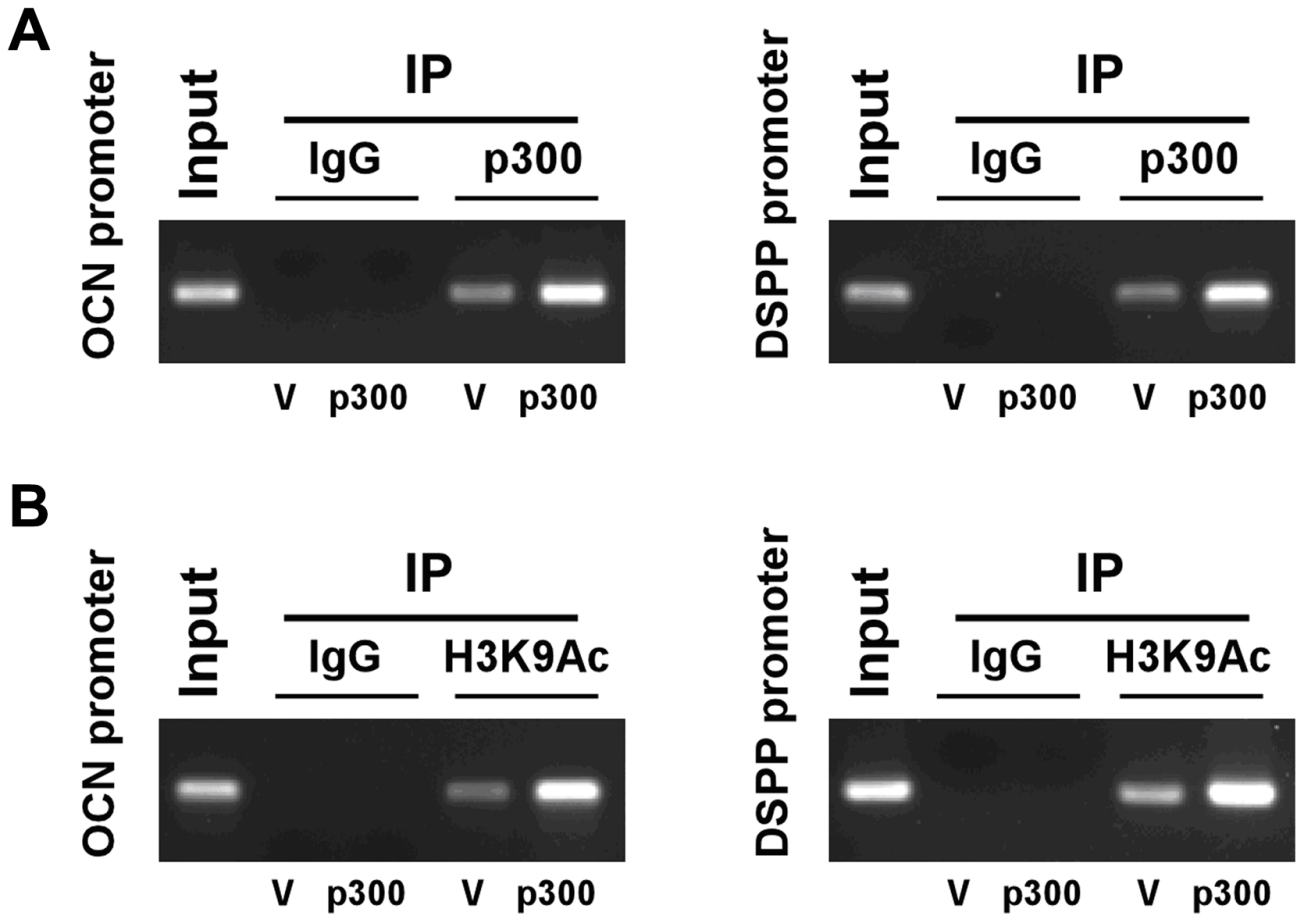
CHIP assay shows that p300 binds to the promoter region of *OCN* and *DSPP* in HDPCs. (A, B) Cells were cross-linked with formaldehyde. Chromatin was immunoprecipitated with anti-p300 or anti-H3K9Ac antibodies. The chromatin was eluted, reverse cross-linked, and the eluted DNA was analyzed by PCR.

To verify whether p300 can be recruited to the promoters of *OCN* and *DSPP* in odontoblastic induction medium, we performed ChIP assays of HDPC/p300 cells. Antibodies against IgG were used as negative controls. The results of this assay showed that anti-p300 but not IgG antibodies immunoprecipitated the *OCN* and *DSPP* promoter in HDPC/p300 cells (Figure 8A). Therefore, p300 was recruited to the promoter region of *OCN* and *DSPP*. We then examined whether p300 can affect the histone acetylation of *DSPP* and *OCN* promoters by ChIP assay using an anti-H3K9Ac antibody. Figure 8B demonstrates that the overexpression of p300 enhanced the level of H3K9Ac in the promoter region of the *OCN* and *DSPP* genes.

## Discussion

p300 is one of several transcriptional coactivators that has been shown to possess intrinsic acetyltransferase activity. p300 acts as a transcriptional adapter for many DNA-binding activators, and its expression has been detected in multiple cell lines. Chen et al. previously reported that p300 expression can be detected during murine tooth development [22]; however, no data on the expression pattern of p300 in human dental pulp cells are available. In this study, we demonstrate that p300 mRNA and protein can be detected in wild-type HDPCs and that p300 levels gradually decrease when the cells are serially passaged. p300 expression decreased when HDPCs were induced to undergo odontogenic differentiation, which might be related to the reduced FBS content of the odontoblastic induction media.

It has been demonstrated that the key pluripotency molecules OCT4, NANOG and SOX2 can cooperatively maintain the regulatory network responsible for self-renewal and pluripotency in ES cells. These pluripotency markers are also expressed in somatic stem cells that have superior expansion and differentiation potential [26]. Previous reports have confirmed that the expression of OCT4, NANOG and SOX2 genes can be detected in dental pulp-derived cells. Although they are expressed at low levels, these genes regulate the “stemness” of dental pulp-derived cells [20], [21].

p300 activity in ES cells is closely linked to the expression of OCT4, NANOG and SOX2, and p300 plays a critical role in the ES cell transcription network [14]. According to a previous study, the expression level of NANOG but not OCT4 was markedly lower in embryoid bodies (EBs) from p300^−/−^ mouse ES cells than in EBs from wild-type ES cells. This finding suggested that p300 is involved in regulating NANOG expression during the differentiation of mouse ES cells and that this regulation most likely occurs through the epigenetic modification of histones near the NANOG-encoding sequence [11]. The interaction between p300 and SOX2 has also been corroborated by *in vitro* studies. p300 can acetylate the DNA-binding domain of SOX2, thus enhancing global acetylation in mouse ES cells. Mimicking acetylation at a key lysine residue also enhances the interaction of SOX2 with the nuclear export machinery [16]. In our study, we transfected HDPCs with expression vectors for the wild-type p300 and its HAT-deletion mutant to determine whether p300 regulates the expression of OCT4, NANOG and SOX2. We found that the overexpression of p300 upregulated the production of *OCT4, NANOG* and *SOX2* mRNA and of NANOG and SOX2 proteins. The protein levels of OCT4 were not significantly altered, perhaps due to post-transcriptional modifications of OCT4. The overexpression of p300 increased the activities of promoter reporter genes associated with *NANOG* and *SOX2*.

We found that the overexpression of a mutant form of p300 lacking the HAT domain in HDPCs resulted in the decreased expression of NANOG and SOX2 mRNA and proteins. The downregulation of NANOG expression by p300-ΔHAT was particularly significant. Li et al. reported that the overexpression of p300 upregulated the promoter activity and the mRNA and protein expression of the human pituitary tumor transforming gene (*hPTTG*). Moreover, the HAT activity of p300 was critical for the regulatory function of this protein [27]. p300 interacts with a series of cardiac-specific genes; however, when the HAT of p300 is inhibited by curcumin, histone acetylation in the promoter regions of these gene does not occur, and the expression of these genes is down-regulated [28]. Our results also showed that the HAT activity of p300 is important for the transcriptional regulation of NANOG and SOX2 in HDPCs. However, the p300-ΔHAT mutant, which lacks the HAT domain, is still able to produce significantly higher levels of these proteins than the control, which has normal endogenous levels of p300. We also showed that p300-ΔHAT was able to stimulate the promoter activities of these genes, albeit at a much lower level. This finding implies that the HAT domain of p300 may partially mediate the regulation of these factors. The upregulation of the expression of these genes and proteins in the p300-ΔHAT mutant cells may be a result of the ability of p300 to indirectly acetylate the relevant histones; however, this upregulation may also be attributable to other functions of p300.

The pluripotency factors NANOG and SOX2 are involved in maintaining the regulatory network responsible for the self-renewal ability and differentiation potential of ES cells and somatic stem cells [10], [29]. Here, we show that the overexpression of p300 in HDPCs upregulates the expression of NANOG and SOX2. We next evaluated whether p300 could induce HDPCs to be less differentiated and whether p300 overexpression is involved in regulating the odontoblastic differentiation potential of HDPCs induced to undergo odontoblastic differentiation. We measured the expression levels of the genes *DMP-1, DSPP, DSP, OCN* and *OPN*, which have been used as mineralization markers for the odontoblast-like differentiation of HDPCs, in HDPC/p300 and HDPC/V cells cultured under normal culture conditions and in odontoblastic differentiation induction medium. DMP-1, a candidate protein for dentinogenesis imperfecta, is present in the extracellular matrix of dentine and bone [30]. DSPP is a major non-collagenous protein found in dentin that is essential for odontoblast differentiation and dentin mineralization [31]. DSP and OCN are considered late markers for odontoblast and osteoblast differentiation [32]. OPN is also mainly expressed in the bone and in dentine [33]. We found that mRNA levels of the odontoblastic differentiation markers *DMP-1, DSPP, DSP, OPN* and *OCN* were significantly decreased in HDPCs overexpressing p300 cultured under normal conditions; this finding suggests that p300 mainly interacted with the “stemness” markers *NANOG* and *SOX2* in non-inductive conditions. Thus, the cells were induced to be more stem-cell-like and therefore less differentiated.

Paino et al reported that valproic acid (VPA), a histone deacetylase (HDAC) inhibitor, did not reduce cell viability or cell proliferation or impact the cell cycle profile of human dental pulp cells; however, VPA did significantly enhance matrix mineralization by increasing OPN and BSP expression [34]. Jin et al demonstrated that Trichostatin A (TSA), another HDAC inhibitor, promoted the proliferation and odontoblast differentiation of HDPCs induced to undergo mineralization differentiation *in vitro* and enhanced dentin formation and odontoblast differentiation *in vivo* during tooth development [35]. The overexpression of p300 did not overtly affect the ability of HDPCs to proliferate in our study. When grown in odontoblastic induction medium, HDPC/p300 cells produced increased levels of *DMP-1, DSPP, DSP, OPN* and *OCN* mRNA, indicating that p300 increased the odontoblast differentiation ability of HDPCs. The enhanced expression of the odontogenic marker genes under inductive medium was different from that under non-inductive medium, which may be related to weakened interactions of p300 with “stemness” genes. These results were further confirmed by the increased ALP activity (an effective early marker of osteoblasts/odontoblasts) observed in HDPC/p300 cells after differentiation induction. Consistent with this finding, alizarin red S staining showed that more mineralized nodules formed at the late stage of odontogenic differentiation in HDPC/p300 cells. However, the p300 mutants lacking the HAT domain induced to differentiate had similarly increased levels of *DMP-1, DSPP, DSP, OPN* and *OCN* mRNA, similarly increased ALP activity and similarly enhanced mineralized nodule formation as the cells with overexpressed p300. Thus, our findings indicate that the overexpression of p300 improves the odontogenic differentiation potential of HDPCs induced to undergo odontoblastic differentiation, and that HAT may not be required for this regulation. We next focused on the mechanism by which p300 regulates the expression of odontogenic differentiation markers. ChIP assays showed that p300 was recruited to the promoter region of *OCN* and *DSPP*, which implies that p300 regulates the expression of odontogenic markers by directly binding to their promoters. The overexpression of p300 also enhanced the level of H3K9Ac in the promoter regions of the *OCN* and *DSPP* genes, indicating that p300 enhanced the histone acetylation of the *OCN* and *DSPP* promoters and thus promoted the transcription of the genes. However, we also showed that the HAT activity of p300 has no significant effect on odontogenic differentiation. p300 has been shown to not only possess acetyltransferase activity but also to interact with numerous transcription factors directly or indirectly through coactivators to stimulate the transcription of specific genes [3], [4]. When p300 is recruited to target genes, other acetyltransferases might also be recruited as coactivators to the same sites to affect the histone acetylation of the target genes. The acetylation of the promoter regions of the *OCN and DSPP* genes may occur due to compensation by other acetyltransferases for the effect of p300. Hence, we inferred that p300 might be acting as a coactivator to regulate the expression of odontoblastic marker genes.

In conclusion, we have shown that p300 is expressed in wild-type HDPCs, that p300 is involved in the upregulation of the key pluripotency molecules NANOG and SOX2 and that the p300 HAT domain is partially required for this upregulation. The mRNA expression of odontoblastic markers, the ALP activity and the formation of mineralized nodules were enhanced when HDPCs overexpressing p300 were induced to differentiate. Thus, our study provides valuable insights into the molecular mechanisms by which p300 regulates the expression of NANOG and SOX2 and the odontogenic differentiation potential of HDPCs. Our findings may have important implications for tooth regeneration studies.

## Materials and Methods

### Ethics Statement

All patients and guardians on behalf of the minors/children who enrolled in our study gave written informed consent for this investigation; the study was approved by the Ethical Review Board of the Guanghua School of Stomatology of Sun Yat-sen University. The investigation also conforms to the principles outlined in the Declaration of Helsinki.

### Cell culture

HDPCs were isolated and cultivated as previously described by Gronthos et al [23]. HDPCs were isolated from impacted third molars extracted for orthodontic reasons from healthy donors aged 17 to 25 years. Only healthy teeth without carious disease or hyperemic pulp tissue were selected. Immediately after extraction, the teeth were washed with 70% ethanol and with phosphate-buffered saline (PBS, pH 7.4). Subsequently, the teeth were cut around the cemento-enamel junction to expose the pulp chamber; the pulp tissue was gently separated from the crown and root using sterile forceps and minced into small pieces. The small fragments were digested for 30 minutes at 37°C in a solution with 3 mg/mL type I collagenase and 4 mg/mL dispase (Gibco, CA, USA). Following digestion, the pulp tissues were placed in 25 cm^2^ culture flasks containing DMEM supplemented with 10% fetal bovine serum (FBS), 100 units/ml penicillin and 100 mg/ml streptomycin (Gibco, CA, USA) and incubated at 37°C in an atmosphere of 95% O2 and 5% CO2. The media were changed every 3 days, and outgrowths from the minced pulp tissue explants were observed after 10 to 14 days of culture. When the cells reached confluence, they were harvested by treatment with 0.25% trypsin with 0.25 mM EDTA (Gibco, CA, USA) and serially passaged for further experiments. Cells from the second or third passage were used for further study.

### Construction and production of plasmids and recombinant lentiviral vectors

The expression vectors containing human wild-type (wt) p300 (pCI-p300) or its HAT-deletion mutant (pCI-p300, HATΔ1472–1522) were generously provided by Dr. Joan Boyes (Institute of Cancer Research, London, UK).

The recombinant lentivirus vectors pCDH-CMV-MCS-EF1-copGFP containing either human p300 or its HAT-deletion mutant sequences were constructed. The empty lentivirus vector pCDH-CMV-MCS-EF1-copGFP was used as a negative control. The identities of all recombinant vectors were confirmed by polymerase chain reaction (PCR) and DNA sequencing. The recombinant lentiviral vectors were produced by transfecting 293T cells. After the 293T cells reached 40% confluence, they were cotransfected with 20 µg of lentivirus vector DNA and packaging vectors composed of 15 µg of psPAX and 5 µg of pMD2.G in serum-free Opti-MEM using Lipofectamine 2000 reagent (Invitrogen Co., Carlsbad, CA, USA) according to the manufacturer's instructions. Six hours after the transfection, the transfection mix was replaced with fresh DMEM supplemented with 10% FBS. The lentivirus-containing culture medium was harvested and filtered through a 0.45 µm protein binding filter 24 and 36 hours post-transfection. The collected viruses were either used immediately or stored at −80°C in 10% FBS for later use.

The *NANOG* and *SOX2* promoter fragments were amplified by PCR using the following primers: *NANOG*: 5′-GTAGAAGGAATGAGAAGACT-3′ (sense) and 5′-TCTTTTAACCACGC TGCACT-3′ (antisense); *SOX2*: 5′-GCTCACTTCCTCTGACTCT- 3′ (sense) and 5′-TGGCAA CAACCAAAACAGT-3′ (antisense). The fragments were cloned into pGL3-Basic vectors (Promega, Madison, WI, USA) between HindIII and BamH1 sites, creating the *NANOG* and *SOX2* promoter/luciferase plasmid.

### Establishment of stable p300/p300-ΔHAT overexpression in HDPCs

Cells were maintained in the culture medium and incubated at 37°C in 5% CO_2_ until they reached approximately 40% confluence. Subsequently, the cells were infected in DMEM with 10% FBS containing the recombinant lentiviral vectors for 4 hours at a multiplicity of infection (MOI) of 50; the supernatant was then replaced with fresh culture medium. Polybrene (8 µg/ml) was added to increase the infection efficiency. Cells were infected with the lentivirus three times over the course of 48 hours. The expression of green fluorescent protein (GFP) detected using fluorescence microscopy (Zeiss, Jena, Germany) showed that approximately 80% of the cells were infected within three days of the transfection. Puromycin (Sigma-Aldrich, St Louis, MI, USA) was added at a concentration of 1.25 µg/ml to the medium for 2 to 4 days to select stably transfected cells.

### Odontoblastic induction of HDPCs

For odontoblastic differentiation, cells were seeded in 24-well and 12-well plates at a density of 2×104 and 5×104 cells per well, respectively, and cultured in DMEM containing 10% FBS until they reached 80% confluence. The media were then replaced with odontoblastic differentiation media containing DMEM supplemented with 5% FBS, 50 µg/ml ascorbic acid, 10 mM b-glycerophosphate and 10^−7^ M dexamethasone (Sigma-Aldrich, St Louis, MI, USA). The culture medium was changed every 3 days.

### Cell proliferation assay

The effect of p300 on the proliferation of HDPCs was examined with the CCK-8 (Cell Counting Kit-8) assay (Dojindo, Kumamoto, Japan) and BrdU assay (Sigma-Aldrich, St Louis, MI, USA) according to the manufacturer's protocol. For the CCK-8 assay, a total of 3×10^3^ cells per well were cultured in 96-well plates at 37°C in 100 µl of DMEM with 10% FBS. After 1, 2, 3 or 4 days of incubation, the supernatant was removed, and 110 µl of DMEM medium containing 10 µl of CCK-8 were added to each well for another 2 hours at 37°C. The optical density (OD) value for each well was read at 450 nm using an automated microplate reader (Sunrise, Tecan, Switzerland).

For the Brdu assay, the HDPSC/v, HDPSC/p300-ΔHAT and HDPSC/p300 cells were labeled with 10 µmol/L BrdU for 4 hours and fixed with 70% ethanol at −20°C overnight. The cells were washed three times in PBS containing 0.5% IFS (Gibco, CA, USA) and treated with 2 mol/L HCl with 0.5% IFS for 20 minutes at room temperature. To stain the nuclei, cells were incubated in serum-free medium with an anti-BrdU antibody (ab152095-Abcam, Cambridge, UK) for 20 minutes at 4°C. A secondary FITC-conjugated rabbit anti-mouse antibody (EarthOx, CA, USA) was applied for another 20 minutes. After extensive washing, the stained cells were counted under a fluorescence microscope (Zeiss, Jena, Germany).

### Real-time quantitative PCR

Total RNA was extracted from dental pulp cells using TRIzol reagent (Invitrogen, Carlsbad, CA, USA) according to the manufacturer's instructions. The extracted RNA was pretreated with RNase-free DNase (Promega, Madison, WI, USA), and 2 µg of RNA from each sample were reverse-transcribed for cDNA synthesis using the RevertAid First Strand cDNA Synthesis Kit (Fermentas, Ontario, Canada) and random primers. Subsequently, the cDNA was used as a template for the PCR. Real-time quantitative RT-PCR was performed using the LightCycler 480 SYBR Green I Master (Roche, Basel, Switzerland) with specific primers according to the manufacturer's instructions. GAPDH was used as a house-keeping gene to normalize gene expression levels. The following reagents were mixed in a final reaction volume of 20 µl: 10 µl of Master Mix, 1 µl of forward and reverse primers, 3 µl of H2O, and 5 µl of cDNA. Cycling conditions were as follows: 1 cycle at 95°C for 3 minutes, followed by 40 cycles at 95°C for 10 seconds, 55°C for 10 seconds, and 72°C for 30 seconds. Relative differences in PCR results were calculated using the comparative ΔΔCt method. Primers were designed using Primer Express v 3.0 software (Applied Biosystems, Inc. Foster City, CA, USA) as follows: *p300*: 5′-GCGGCCTAAACTCTCATCTC-3′ (sense) and 5′-TCTGGTAAGTCGTGCTCCAA-3′ (antisense); *NANOG* 5′-GATTTGTGGGCCTGAAGAAA-3′ (sense) and 5′-ATGGAGG AGGGAAGAGGAA-3′ (antisense); *OCT4*: 5′-GTGGAGGAAGCTGACAACAA-3′ (sense) and 5′-GGTTCTCGATACTGGTTCGC-3′ (antisense); *SOX2*: 5′-GCTTAGCCTCGTCGATG AAC-3′ (sense) and 5′-GCTTAGCCTCGTCGATGAAC-3′ (antisense); *DMP-1*: 5′-TGGGCA TAGATTTCCTCTTTG-3′ (sense) and 5′-TGAGCAGGATGCTGATCTTC-3′ (antisense); *DSPP*: 5′-GCCACTTTCAGTCTTCAAAGAGA-3′ (sense) and 5′-GCCCAAATGCAAAAATATG TA-3′ (antisense); *DSP*: 5′-CAGGATGTACTATTCTCGGCG-3′ (sense) and 5′-GTGTTCT GGTTCTGGTGCCT-3′ (antisense); *OCN*: 5′-CTCACACTCCTCGCCCTATT-3′ (sense) and 5′-TTGGACACAAAGGCTGCAC-3′ (antisense); *OPN*: 5′-GTGATTTGCTTTTGCCTCCT-3′ (sense) and 5′-GCCACAGCATCTGGGTATTT-3′ (antisense); *GAPDH*: 5′-AAGGTGAA GGTCGGAGTCAA-3′ (sense) and 5′-AATGAAGGGGTCATTGATGG-3′ (antisense).

### Western blotting

Cells were harvested in protein lysis buffer (50 mM Tris, 150 mM NaCl, 1% Triton X-100, 1% sodium deoxycholate, 0.1% SDS, sodium orthovanadate, sodium fluoride, EDTA) containing protease inhibitors (Beyotime, Haimen, China) and incubated on ice for 30 minutes. Protein concentrations were measured with a bicinchoninic acid (BCA) protein assay (Beyotime, Haimen, China). Thirty micrograms of protein were separated by 10% sodium dodecyl sulfate- polyacrylamide gel electrophoresis (SDS-PAGE) and electrophoretically transferred onto a polyvinylidene fluoride (PVDF) membrane (Millipore, Billerica, MA, USA). The membrane was incubated for 1 hour at room temperature in TBST containing 5% skim milk to block nonspecific protein binding and incubated at 4°C overnight with the primary antibodies anti-p300 (ab54984-Abcam, Cambridge, UK; 1∶200), anti-NANOG (ab62734-Abcam, Cambridge, UK; 1∶1000), anti-OCT4 (ab19857-Abcam, Cambridge, UK; 1∶500), anti-SOX2 (ab97959-Abcam, Cambridge, UK; 1∶500) and anti-GAPDH (ab8245-Abcam, Cambridge, UK; 1∶3000). After washing, the membrane was incubated for 1 hour with an HRP-conjugated secondary antibody (ab6728-Abcam, Cambridge, UK; 1∶1000) at room temperature. Antibody binding was visualized with an enhanced chemiluminescence system (Millipore ECL Western blotting Detection System, Millipore, Billerica, MA, USA), and band densities were obtained and normalized to GAPDH and the background using ImageJ.

### Luciferase reporter assay

The HDPCs were seeded in 24-well plates and cultured for 24 hours. The HDPCs were then co-transfected with the promoter/luciferase plasmid and the pCI-p300 plasmid or the HAT-deletion mutant (pCI-p300, HATΔ1472–1522) plasmid using Lipofectamine 2000 reagent (Invitrogen); the empty plasmid pCI was used as a negative control. Cells were incubated at 37°C for 5 to 8 hours, after which the culture medium was changed. Cells were harvested after another 24 hours. Cell lysates were collected and the luciferase activity was measured with a Turner Designs TD-20/20 Luminometer in the Dual-Luciferase Assay System (Promega). A second luciferase gene from Renilla reniformis provided constitutive activity as an internal control.

### Alkaline phosphatase activity analysis

Cells were seeded at a density of 2×10^4^ cells per well in 24-well plates and cultured in DMEM with 10% FBS until they reached 80% confluence; the cells were then cultured in odontoblastic differentiation medium for 0 and 3 days. The medium was replaced every 3 days. Cells were washed three times with PBS; 100 µl of 1% Triton X-100 were then added to each well and the plates were incubated at 4°C overnight. An ALP assay kit (Nanjing Jiancheng Bioengineering Institute, Nanjing, China) was used to analyze ALPase activity according to the manufacturer's instructions. The absorbance was measured at 520 nm using an automated microplate reader (Sunrise, Tecan, Salzburg, Switzerland). The protein content was quantified using a BCA protein assay (Beyotime, Haimen, China). The amount of ALP in the cells was normalized against the total protein content.

### Alizarin red S staining

To assess *in vitro* mineralization, cells were seeded in 12-well plates and cultured in DMEM with 10% FBS until they reached 80% confluence; the cells were then cultured in the differentiation medium for 21 days. After culture, the cells were washed three times with PBS (pH 7.4), fixed in a 4% paraformaldehyde solution for 15 minutes, and washed three times with deionized water. Cells were then incubated in 1% Alizarin red S for 30 minutes, washed 5 times with distilled water, and dried at room temperature. The mineralized nodules were photographed using an inverted microscope (Zeiss, Jena, Germany).

### Chromatin immunoprecipitation assays (ChIP)

HDPC/p300 and HDPC/V cells were cultured in odontoblastic differentiation medium for 7 days. The cells were then fixed by adding formaldehyde at a final concentration of 1% and incubated for 10 minutes at 37°C. Glycine was added to the samples to terminate the cross-linking reaction. The samples were incubated for 5 minutes at room temperature, lysed with SDS lysis buffer and protease inhibitor, and sonicated to generate 500–1000 bp DNA fragments. Agarose gel electrophoresis was used to confirm that the sonification had produced the appropriate degree of shearing of chromatin. Cross-linked proteins were immunoprecipitated using antibodies against p300 (ab14984-Abcam, Cambridge, UK), H3K9Ac (ab10812-Abcam, Cambridge, UK) and IgG (ab2410-Abcam, Cambridge, UK). Complexes were washed in low-salt, high-salt and LiCl buffers once and in a TE buffer twice. The DNA was then extracted and precipitated. To remove the protein-DNA cross-links, NaCl was added to the samples and the samples were incubated overnight at 65°C. The eluates were diluted with ChIP dilution buffer and underwent a secondary immunoprecipitation reaction. To digest the isolated protein, 10 µl of 500 mM EDTA, 20 µ l of Tris HCl (pH 7.2), and 1 µl of proteinase K were added to each sample; the samples were then incubated for 1 hour at 45°C. The enrichment of the DNA template was amplified by PCR using primers designed to specifically amplify the *OCN* and *DSPP* promoter region containing p300 binding sites. The following primer sequences were used: *OCN*: 5′-CTTTGGCTGGCAGTCCCT-3′ (sense) and 5′-GCTTGCTCTTCCC TCCTCT-3′ (antisense); *DSPP*: 5′-GGCCACTTTCAGTCTTCA′ (sense) and 5′-ATCAAACTGG CTTCATCT-3′ (antisense).

### Statistical analysis

Each experiment was performed in triplicate and repeated at least three times. Experimental groups were compared using one-way analysis of variance (ANOVA) or repeated measures ANOVA with SPSS13.0 software (SPSS, Chicago, IL, USA). Values are expressed as the means ± SD. All P-values are two-tailed and *P*<0.05 was considered statistically significant.
